# Feasibility of Systemically Applied dsRNAs for Pest-Specific RNAi-Induced Gene Silencing in White Oak

**DOI:** 10.3389/fpls.2022.830226

**Published:** 2022-03-16

**Authors:** Zachary Bragg, Lynne K. Rieske

**Affiliations:** Department of Entomology, University of Kentucky, Lexington, KY, United States

**Keywords:** RNA interference, gene silencing, translocation, tree protection, forest pest management, white oak

## Abstract

The efficacy of double-stranded RNA (dsRNA) in inducing host specific gene knockdown and mortality has been demonstrated in a multitude of insects and dsRNAs are being integrated for pest suppression in a variety of agricultural and horticultural crops. However, less attention has been applied to their use in forest settings, despite the demonstrated susceptibility of multiple forest pests to RNAi. Prior to implementation for forest pest suppression, characterization of the specificity, efficacy, and behavior of dsRNAs in the environment is essential. Therefore, we investigated the translocation and retention of exogenously applied dsRNA in an economically and ecologically significant hardwood tree when applied hydroponically. White oak (*Quercus alba*, L.) seedlings were exposed to dsRNAs as a root soak, and at 1, 3, 5, and 7 days post-exposure were destructively sampled, divided into stem and leaf tissue, and the RNA extracted. Gel electrophoresis was used to visualize the presence of exogenous dsRNA in treated seedling material and Sanger sequencing was used to further verify recovery of treatment dsRNAs. Both techniques confirmed the presence of the exogenously applied dsRNAs in each tissue type at each sample interval, demonstrating successful uptake and translocation of dsRNAs through white oak tissues. Our findings support root uptake as a viable delivery method for dsRNAs in hardwood seedlings, which could provide single tree protection from selected tree feeding pests or pathogens.

## Introduction

Recent advances in molecular technologies have enabled multiple novel pest management approaches, including the adaptation of RNA interference (RNAi) technologies for agricultural and horticultural commodities. RNAi is a molecular, anti-viral pathway that evolved in eukaryotic cells prior to the divergence of plant and animal lineages, making this mechanism present across a diverse array of organisms (Sharp, 2001). The RNAi pathway can be induced by a variety of natural or synthetic genetic sequences including small-interfering RNA (siRNA), micro-RNA (miRNA), short hairpin RNA (shRNA), and double stranded RNA (dsRNA). The ability to manipulate these pathways has led to a revolution in functional genomics, biotechnology, and genetic engineering in plants, animals, and fungi (Kamath et al., 2003; Small, 2007; Shafran et al., 2008). In addition to its utility in elucidating gene function, the ability of RNAi to induce post-transcriptional gene silencing leading to gene knockdown makes it a potentially powerful management tool for controlling viruses (Worrall et al., 2019), fungi (Rosa et al., 2018), nematodes (Fairbairn et al., 2007), and insects (Yu et al., 2013). A conserved antiviral pathway, RNAi is a stepwise process initiated when the enzyme Dicer cleaves exogenous dsRNAs into 21–23 bp RNAs called small-interfering RNAs. These double-stranded siRNAs are then unwound into single strands, only one of which, the guide strand, binds to the protein complex RISC (RNA-induced silencing complex) that acts as a guide, binding to complementary strands of messenger-RNA (mRNA) transcripts. Once bound, Argonaute, a protein component in RISC, degrades the complementary mRNA, leading to translational inhibition (Zamore, 2001). By engineering dsRNA sequences complementary to specific genes in pest species, the RNAi pathway can be induced to initiate targeted gene silencing of critical processes or specific biological functions. One advantage of RNAi technology over traditional insecticides is its specificity to target, in where an exact match of ≥16 base pairs is required to cause gene silencing (Chen et al., 2021). Both *in silico* and *in vivo* studies have demonstrated reduced off target effects that RNAi technologies offer when compared to traditional insecticides (Vogel et al., 2019; Pampolini and Rieske, 2020).

Since its discovery in the nematode *Caenorhabditis elegans* (Maupas) (Fire et al., 1998) dsRNA induced gene silencing has been demonstrated in a diverse array of organisms, including the arthropod lineages of Coleoptera, Lepidoptera, Diptera, Hemiptera, Blattodea, and Acari (Kunte et al., 2020). In laboratory and field settings dsRNAs and other RNAi constructs have been deployed and successfully demonstrated gene knock down when applied topically (Miguel and Scott, 2015), systemically (Dalakouras et al., 2018), and transgenically, *via* host plant expression (Baum et al., 2007; Fu et al., 2020). In agriculture deployment of RNAi technology has moved beyond the theoretical framework, when in 2017 the US Environmental Protection Agency began the process of approving the first genetically engineered dsRNA expressing crop, SmartStax^®^ PRO (Bayer, Leverkusen, Germany), which combines glyphosate resistance, traditional *Bacillus thuringiensis* protection, and novel RNAi technology targeting the highly destructive western corn rootworm (*Diabrotica virgifera*, LeConte) (Head et al., 2017). While numerous agricultural and horticultural pests have been the focus of efforts to develop RNAi technologies for plant protection, considerably less attention has been given to shade tree and forest pests.

Susceptibility to RNAi induced gene knockdown and mortality has been demonstrated in numerous deciduous and coniferous tree-feeding pests, including the emerald ash borer (*Agrilus planipennis*, Fairmaire) (Zhao et al., 2015; Rodrigues et al., 2018), the Asian longhorned beetle (*Anoplophora glabripennis*, Motschulsky) (Rodrigues et al., 2017), and the southern and mountain pine beetles (*Dendroctonus frontalis*, Zimmermann, and *Dendroctonus ponderosae*, Hopkins) (Kyre et al., 2019; Kyre et al., 2020). In addition to proofs of concept, identification of effective target genes, and demonstrations of specificity (Pampolini and Rieske, 2020), investigations into options for RNAi delivery in woody plants are under way (Pampolini et al., 2020). The domesticated fruiting apple tree (*Malus domestica*, Borkh) and the woody grape vine (*Vitis vinifera*, L.) retain and translocate hairpin RNAs applied through trunk injection (Dalakouras et al., 2018), and large doses of dsRNA applied as a root drench to 3 m tall *Citrus* sp. trees can be recovered within plant tissues 57 days post-exposure (Hunter et al., 2012). These findings support the concept of individual tree protection using RNAi based technologies. However, a more thorough understanding of the spatial and temporal behavior of these dsRNAs in various plant tissues is crucial. Therefore, we applied a root soak of dsRNA to white oak (*Quercus alba*, L.) seedlings to better understand tissue specific (i) translocation and (ii) persistence within our model deciduous tree species. We sought to evaluate the presence and persistence of exogenously applied dsRNA within white oak tissues as a means of validating RNAi technology for tree protection.

## Materials and Methods

### Plant Material

Bareroot, 1-0 white oak seedlings (Kentucky Division of Forestry, West Liberty, KY, United States) were lifted from nursery beds in March 2020 and planted directly into Promix general purpose growing medium BX (Premier Tech Horticulture, Rivière-du-Loup, QC, Canada) in 6.35 × 25.40 cm D40H deepot tree growing cells (Stuewe & Sons Inc., Tangent, OR, United States). Seedlings were maintained in the greenhouse (∼18–22°C, 15:9 L:D) and watered daily through leaf flush, and then as needed.

### Target Gene Selection and dsRNA Synthesis

To date no target genes and corresponding dsRNA primers have been developed for pests of white oak, consequently we used a non-pest specific transcript in this experiment. Green fluorescent protein (*gfp*) is not present in insects and as such is widely used as a negative control for insect gene expression studies (Nunes et al., 2013); therefore double-stranded *gfp* (dsGFP) was utilized as our dsRNA treatment.

To amplify dsRNA template transcripts, polymerase chain reactions (PCR) were run using *gfp* purified PCR product as template and primers specific to the gene of interest (Table 1) using the following parameters: 4 min at 94°C, followed by 35 cycles of 30 s at 94°C, 30 s at 60°C, and 45 s at 72°C, and a final incubation step at 72°C for 10 min. PCR products were purified using a Qiagen purification kit according to the manufacturer’s instructions (Qiagen, Germantown, MD, United States). Following transcript purification, the MEGAscript RNAi Kit (Thermo Scientific, Waltham, MA, United States) was used to synthesize dsRNA. This reaction mixture was incubated at 37°C for 16 h. Following incubation, dsRNA was recovered by adding 0.1 × volume sodium acetate and 2.4 × volume of 100% ethanol and incubating at −20°C for 2 h. After incubation, the mix was centrifuged (20,000 × *g*) at −4°C for 30 min, washed with 750 μL of 75% EtOH, and centrifuged again (18,800 × *g*) at -4°C for 15 min. After the ethanol wash, samples were dried at 37°C for 25 min and re-suspended in 20 μL of nuclease free H_2_O. Quality of dsRNA was checked using electrophoresis and quantified with a spectrophotometer (NanoDrop Technologies, Wilmington, DE, United States) (Supplementary Table 1 and Supplementary Figure 1).

**TABLE 1:** Primer sequences for PCR amplification and dsRNA synthesis.

Gene	Type	Primer sequence 5’3’		Amplicon (bp)
*gfp*	PCR/dsRNA	**F**	**TAATACGACTCACTATAGGG** CGATGCCACCTACGGCAA	288
		**R**	**TAATACGACTCACTATAGGG** TGTCGCCCTCGAACTTCA	
*rps3*	PCR	**F**	**TAATACGACTCACTATAGGG** CATGGGTAAGGATAGGGTAATG	439
		**R**	**TAATACGACTCACTATAGGG** GCTATTTCTGCGCCTTCT	

*Promoter sequence of T7 RNA polymerase in bold. Expected amplicon size, including T7 sequence, listed in base pairs.*

### dsRNA Exposure

**FIGURE 1:**
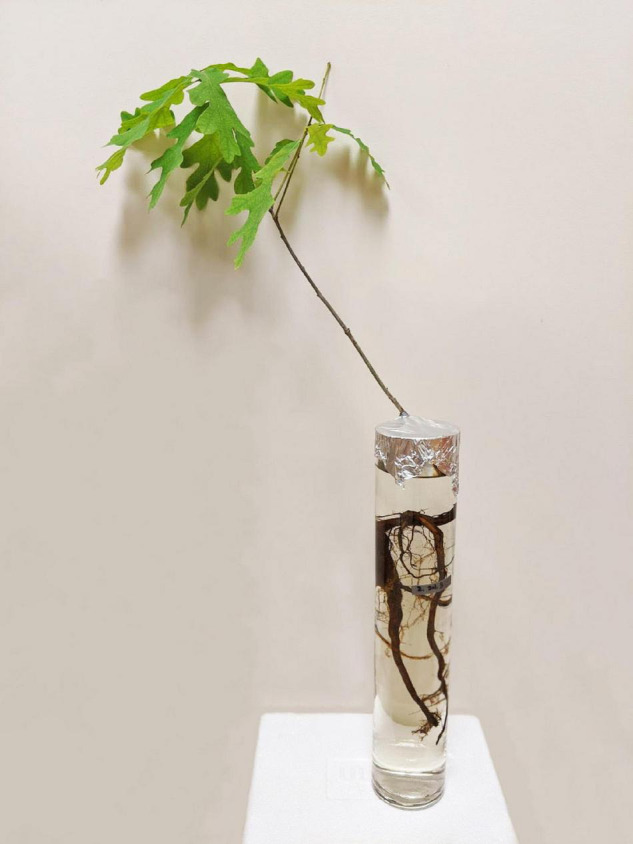
dsRNA solution was pipetted onto wall of glass assay cylinder for each treatment seedling. Containers were filled with 1.8 mL dd H_2_O after which seedlings were submerged to the root collar.

For dsRNA exposure, seedlings were gently removed from potting medium and the roots were rinsed with tap water, followed by rinsing for 30 s with dd H_2_O before transferring to autoclaved 1.85 L clear glass assay cylinders (7.5 cm × 40.5 cm). Seedlings were randomly assigned to receive either 16 μg dsGFP (*n* = 12), or the control, which received no dsRNA (*n* = 3). dsRNA treatments were pipetted onto the wall of each assay cylinder, after which dd H_2_O was added to achieve a total volume equal to 1.8 L with root volume displacement. dsRNA synthesis for each replicate was conducted simultaneously, however concentrations of each dsRNA mixture and therefore subsequent treatment volumes varied between replications (Supplementary Table 1). dsGFP treated seedlings were randomly assigned to exposure intervals of 1, 3, 5, and 7 days (*n* = 3), while control seedlings (–dsRNA) were assigned to a 9 days exposure interval (*n* = 3). Control seedlings were only used during replicates 1 and 2 due to limited seedling availability. Assay cylinders were topped with aluminum foil sheets to minimize water loss due to evaporation (Figure 1). Seedlings were maintained in greenhouse conditions (∼18–22°C, 15:9 L:D) and rotated daily to minimize any abiotic irregularities. Nuclease free dd H_2_O was also added daily to maintain a total volume of 1.8 L. Replicates 1 and 2 (*n* = 15, each) were completed in July and replicates 3 and 4 (*n* = 12, each) were completed in August 2020.

### Plant Processing

#### Tissue Segmentation and Homogenization

At each interval, designated seedlings were removed from assay cylinders and rinsed with dd H_2_O for 30 s to remove any treatment material. Total seedling length from the tip of the tap root to the apical meristem [hereafter simply “height” (cm)] and root collar diameter (RCD) (mm) were measured. Next, seedlings were sectioned into (*i*) new growth consisting of stem tissue generated during the current growing season, designated as “stem” tissues, and (*ii*) foliage with leaf petioles included. Sectioning tools were sterilized with dilute bleach solution between each tissue sample. Tissues were ground to a fine powder using liquid nitrogen and a mortar and pestle, and ∼200 mg of each tissue type was transferred to a 1.5 mL microcentrifuge tube and stored at −80°C until RNA extraction. A two-way ANOVA with type II SS was used to evaluate seedling size; Tukey’s HSD was used to determine differences in seedling height and RCD for each exposure interval and between each replicate. Seedlings designated as controls were not included in statistical analyses evaluating seedling size.

#### RNA Recovery and Quantification

Following tissue homogenization, total RNA was extracted using protocols adapted from Chang et al. (1993) (Supplementary Text 1). RNA pellets were resuspended in 20 μL RNase free H_2_O for 30 min at 21°C with 5 s of light vortexing every 10 min. Once completely resuspended, RNA concentration and integrity were analyzed *via* absorbance measurements of 260/280 nm and 260/230 nm (NanoDrop Technologies, Wilmington, DE, United States). Concentration of each sample was recorded in ng/μL and absorbance measurements were recorded as ratios. RNA samples were then stored at −20°C until cDNA synthesis. For dsRNA treated samples there were four exposure intervals (1, 3, 5, and 7 days), each with three biological replicates, and two tissue types (stem and foliage), generating 96 RNA samples across all four replicates. A two-way ANOVA with type II SS was used to evaluate total RNA recovery; Tukey’s HSD was used to determine differences between tissue types, exposure interval, and replicate. Initial analyses included all samples, however RNA samples from seedlings designated as controls were not included in final statistical analyses, as control seedlings were used only to verify the absence of treatment dsRNAs in untreated tissue.

### Analysis of dsRNA Presence

#### cDNA Synthesis and Polymerase Chain Reactions Amplification

Following RNA recovery, complementary-DNA (cDNA) was synthesized using a template of 1,000 ng of RNA, unless the initial concentration of RNA was not suitable (1 of 108 samples), in which case the maximum template size was used. Each cDNA sample corresponded to a unique combination of tissue type (stem or foliage), dsRNA treatment (dsGFP or –dsRNA), and exposure interval (1, 3, 5, 7, or 9 days). Samples had one PCR completed using primers corresponding to the exogenously applied dsGFP treatment, and a second PCR with primers corresponding to an endogenous control gene. The gene encoding ribosomal protein S3 (*rps3*) is integral to ribosomal structure and is found throughout oak tissue types and was thus selected as an endogenous control to verify the success of RNA extraction, cDNA synthesis, and PCR amplification. Both transcripts were amplified from the cDNA using PCR protocol with modified incubation parameters consisting of an extended cycle count: 4 min at 94°C, followed by 40 cycles of 30 s at 94°C, 30 s at 60°C, and 45 s at 72°C and a final incubation step at 72°C for 10 min.

#### Gel Electrophoresis and Visualization

After amplification, samples were prepped for visualization by combining 7.5 μL of PCR product with 2.5 μL of SmartGlow Loading Dye (Stellar Scientific, Baltimore, MD, United States), vortexing for 10 s, and pipetting into wells in a 1% agarose gel and run for 60 min at 115 v. Products were visualized using an UV Transilluminator and photographed; presence of an amplicon matching the size of the treatment dsRNA equated to successful recovery of exogenous dsRNA in each tissue type. Differences in recovery by tissue type were assessed with Pearson’s chi-squared test.

To quantify results we assigned presence/absence values to each sample. Presence was confirmed by presentation of a distinct product band matching the size of the sequenced pre-treatment products; absence was assigned to samples lacking a distinct band or ones having smeared bands. As a result of primer design, both *gfp* and *rps3* samples formed non-specific products which were simply ignored as these were not vital to our experimental question but merely artifacts of primer dimerization. Instead, only the desired products, as confirmed by sequencing and product size matching, were counted and analyzed.

Based on gel images, dsRNA recovery for each sample was treated as a binary dependent variable, with successful recovery equal to 1 and unsuccessful recovery 0. A logistic regression model was selected to estimate the factors which influence successful recovery of dsRNA in treated tissues. Separate models were created for each tissue type, where in each recovery of exogenous dsRNA served as the response variable and RCD, height, exposure interval, and recovered RNA concentration all served as predictors and were considered continuous variables. Models were compared using Efron’s pseudo *R*^2^ and the Akaike information criterion (AIC) and individual parameters were assessed using Pearson’s chi-squared test.

#### Product Sequencing

Treatment and recovered genetic materials were Sanger sequenced to confirm the successful amplification of exogenously applied dsRNA from woody tissue samples. A subset of samples (cDNA) from the previous analyses were amplified using PCR to generate adequate sample quantities for subsequent gel extraction and purification. Following initial PCR amplification, each new sample was run on a gel and the desired products were extracted from agar using QIAquick Gel Extraction Kit (Qiagen, Germantown, MD, United States). Gel extraction served to separate desired products from undesired non-specific products associated with both *gfp* and *rps3* primer designs. Once extracted and purified, an additional PCR was run to obtain ample material for genetic sequencing. PCR samples (Table 2) were sent to the University of Kentucky Health Center Genomics Core Laboratory (Lexington, KY, United States) for reaction preparation, clean up, and sequencing. Sequences from treatment and recovered samples were compared to verify the presence of exogenously applied treatment material. Pairwise nucleotide sequence alignment for taxonomy tool (EzBioCloud, ChunLab Inc., Republic of Korea, 2017) was used to align forward and reverse reads from treatment and recovered pairs, and similarity scores based on Myers and Miller global alignment algorithm were used to verify the presence of treatment material in post-treatment tree tissue.

**TABLE 2:** Sequenced amplicons from dsRNA synthesis and recovered seedling material.

Amplicon name	Description
treatment-GFP	PCR product used as template to make dsGFP
recovered-GFP	PCR product amplified using dsGFP primers on stem tissue from seedling treated with dsGFP
reference-GFP	Sequence for *gfp* amplicon made using dsGFP primers
control-RPS	PCR product amplified using *rps3* primers on stem tissue from seedling treated with dsGFP

## Results

### Plant Material and RNA Recovery

Seedling size did not differ between treatments (dsGFP vs. –dsRNA), replicates, or dsRNA exposure intervals. Treated seedlings across all replicates (*N* = 48) had a RCD of 6.32 mm ± 0.259 (X̄ ± SE) and a height of 72.3 cm ± 2.12; neither RCD [*F*_(3_,_44)_ = 0.618, *p* > 0.05] nor height [*F*_(3_,_44)_ = 0.071, *p* > 0.05] differed between replicates, nor did they differ between dsRNA exposure intervals [RCD: *F*_(3_,_44)_ = 0.547, *p* > 0.05; height: *F*_(3_,_44)_ = 0.150, *p* > 0.05].

Across all four replicates the average RNA concentration was 324.8 ng/μL ± 14.4 (∼6,496 ng total). RNA yield differed significantly between replicates [*F*_(3_,_92)_ = 9.86, *p* < 0.001]; a *post hoc* Tukey test revealed that replicates 1, 2, and 3 all shared similar yields and replicate 4 showed significantly higher recovery due perhaps to increased efficiency in extraction protocol (rep 1: 260.0 ng/μL; rep 2: 285.0 ng/μL; rep 3: 314.4 ng/μL; rep 4: 439.9 ng/μL). Despite the difference in replicate 4, RNA yield did not differ between exposure intervals [*F*_(3_,_92)_ = 1.23, *p* > 0.05] or between tissue types [*F*_(1_,_94)_ = 2.18, *p* > 0.05]. Additionally, RNA purity was consistent across replicates, with none of the 96 samples showing absorbance < 1.75 at 260/280 nm. However, ∼ 64% of our samples (61 of 96) had 260/230 nm absorbance readings < 1.75, indicating the presence of a non-nucleic acid component, perhaps due to high phenolic concentrations in oak tissues which can be difficult to remove during RNA extraction (Newbury and Possingham, 1977).

### Recovery of Exogenous dsRNA

#### Sequence Alignment

Similarity scores based on Myers and Miller global alignment algorithm were used to compare both forward and reverse reads of the treatment and recovered genetic material (Table 3). Percent match was calculated by dividing total matching base pairs by total aligned base pairs, excluding both non-aligned and trailing sections. Additionally, both treatment-GFP and recovered-GFP sequences were aligned to the reference-GFP sequence to control for any sequencing inconsistency. Between the forward and reverse reads, treatment- and recovered-GFP had a total of 78 and 22 single base pair differences respectively and averaged 85.26% match. When aligned to the reference sequence (reference-GFP) treatment-GFP had an average match of 88.71%, whereas recovered-GFP had an average match of 87.02%.

**TABLE 3:** Sequence alignment data for treatment and recovered product pairs.

Read	Sequence 1	Length	Sequence 2	Length	Matches	Errors	Total	Match
F	treatment-GFP	524	recovered-GFP	383	298	78	376	79.26%
R	treatment-GFP	255	recovered-GFP	255	230	22	252	91.27%
F	reference-GFP	248	treatment-GFP	524	205	23	228	89.91%
R	reference-GFP	248	treatment-GFP	255	203	29	232	87.50%
F	reference-GFP	248	recovered-GFP	383	202	31	233	86.70%
R	reference-GFP	248	recovered-GFP	255	200	29	229	87.34%
F	control-RPS-1	455	control-RPS-2	461	431	24	455	94.73%
R	control-RPS-1	404	control-RPS-2	402	387	15	402	96.27%

#### Gel Recovery

**FIGURE 2:**
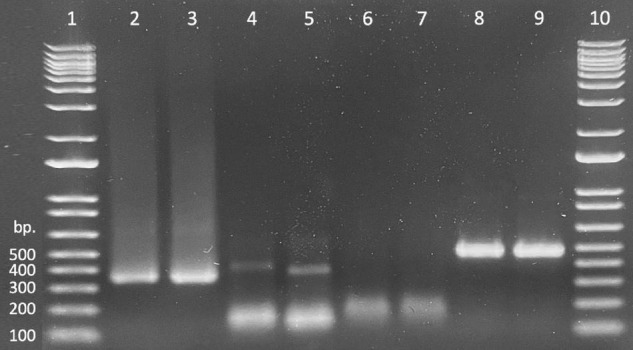
Gel demonstrating recovery of treatment material (dsGFP, lanes 2 and 3), PCR samples amplified from dsGFP treated oak tissues using dsGFP primers (lanes 4 and 5), PCR samples amplified from water treated oak tissues using dsGFP primers (lanes 6 and 7), and samples amplified from dsGFP treated oak tissues using *rps3* primers (lanes 8 and 9). Lanes 1 and 10 contain 1 Kb ladder.

Presence/absence of exogenously applied treatment dsRNAs in seedling tissues was verified by the presence of a band (recovered product) matching the size of the treatment product (Figure 2 lanes 2 and 3). Bright, distinct products as well as faint, but verifiable bands correlated to “presence” or successful recovery (Figure 2 lanes 4 and 5), whereas smeary, inconsistent, or complete lack of bands correlated to “absence” or unsuccessful recovery (Figure 2 lanes 6 and 7). Non-specific products were present in nearly all samples; these were ignored as they did not impede presentation of desired products or interpretation of results. Tissues from untreated control seedlings in reps 1 and 2, exposed to water only (–dsRNA), showed successful amplification of the endogenous control sequence (*rps3*) but no amplification of the treatment (dsGFP) (Figure 2 lanes 8 and 9).

dsRNA was successfully recovered from both tissue types consistently across each of the four exposure intervals and repeatably across all four replicates. Proportional success of dsRNA recovery (no. samples with verified dsRNA recovery divided by total no. samples) slightly decreased as exposure time increased; however, recovery across time points did not differ statistically [χ^2^_(3_,_96)_ = 1.695, *p* = 0.64]. Additionally, recovery between tissue types differed slightly across the time scale, but a chi-square test of independence again showed that there was no significant association between recovery of treatment material and seedling tissue type [χ^2^_(1_,_96)_ = 0.6164, *p* = 0.43]. Overall recovery of exogenously applied dsRNA was 93% successful (Table 4).

**TABLE 4:** Recovery of dsGFP in oak seedling tissues at 1, 3, 5, and 7 days post-treatment represented by percentage and count.

	1 day	3 days	5 days	7 days	All time points
Stem	91.7% _(11/12)_	91.7% _(11/12)_	91.7% _(11/12)_	83.3% _(10/12)_	89.6% _(43/48)_
Foliage	100.0% _(12/12)_	100.0% _(12/12)_	91.7% _(11/12)_	91.7% _(11/12)_	95.8% _(46/48)_
Both tissue types	95.8% _(23/24)_	95.8% _(23/24)_	91.7% _(22/24)_	87.5% _(21/24)_	92.7% _(89/96)_

#### Logistic Regression Modeling

**FIGURE 3:**
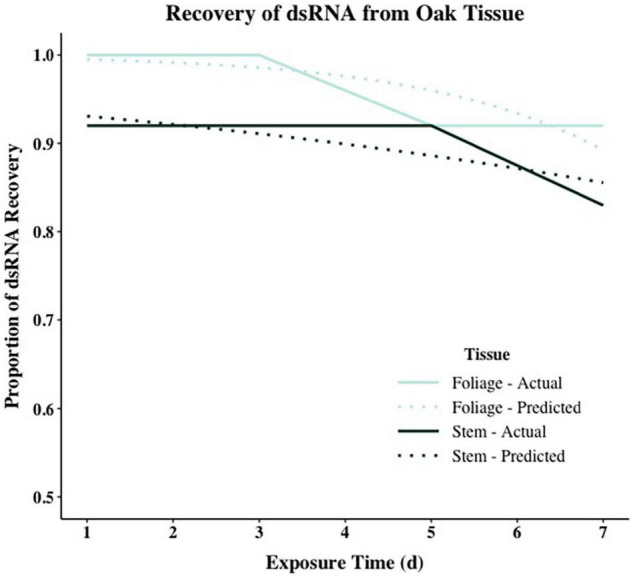
Logistic regression models showing dsRNA recovery in dsGFP-treated foliage and stem tissues of oak seedling across replicates showing actual (solid lines) and projected probabilities (dotted lines). Refer to Table 5 for model statistics.

Model comparison using Efron’s Pseudo *R*^2^ and AIC support different model composition. For both tissue types, models including all predictors (RCD, height, exposure interval, and RNA recovery) had the largest Pseudo *R*^2^ values when compared to all other models in stepwise comparisons. However, using AIC as a comparative metric, the null model containing no predictors had the lowest AIC for stem tissue, while the model including height and time had the lowest AIC for foliar tissue (Supplementary Table 2). When assessing the significance of each model parameter individually, none of the parameters were significant for stem tissue and only height was significant for foliar tissue [χ^2^_(1_,_47)_ = 6.2863, *p* < 0.05] (Supplementary Table 3). Despite accounting for less overall variance in dsRNA recovery, because of their significantly lower AIC values the null model and the model containing height and time were selected as the final models for stem and foliar tissues, respectively (Figure 3 and Table 5). Height was the only significant predictor for foliar tissue, and the odds ratio was <1.0 indicating a negative relationship between increasing height and successful recovery of exogenously applied dsRNA in foliar tissues (Table 5). Interestingly, time, or exposure interval, was not statistically significant as a predictor in any of the models tested.

**TABLE 5:** **(A)** Output of logistic regression model predicting dsRNA recovery in stem tissues. **(B)** Output of logistic regression and **(C)** analysis of deviance table (type III SS) for model predicting dsRNA recovery in foliar tissues.

(A)	Coefficient	Std. Error	*z*-value	Pr (>| z|)
Intercept	2.15	0.473	4.55	5.26 E-6

**(B)**	**Coefficient**	**Std. Error**	***z*-value**	**Pr (>| z|)**

Intercept	33.4	19.9	1.67	0.09
Height	−0.289	0.186	−1.55	0.12
Time	−0.041	0.032	−1.28	0.20

**(C)**	**LR Chisq**	**Df**		**Pr (>Chisq)**

Height	7.09	1		0.008
Time	2.67	1		0.102

## Discussion

Here we demonstrate successful uptake, translocation, and retention of dsRNAs applied exogenously as a root soak in seedlings of white oak, an economically and ecologically significant woody plant, furthering our understanding of how RNAi could be deployed as a pest suppression strategy in a deciduous tree. While extensive research has moved RNAi biopesticides from laboratory experimentation to real world deployment for agricultural pests, this technology has yet to be deployed against woody tree pests. As there are currently no validated target genes for insect pests of white oak, dsGFP was selected here to characterize the spatial and temporal retention of dsRNAs in white oak tissue. While not pest specific, these findings broaden our understanding of naked dsRNAs applied directly to plant roots and can serve as a model for larger scale experiments aimed to bridge the gap between laboratory technology and practical deployment. A thorough understanding of dsRNA movement and persistence in woody tissues is essential before RNAi can be efficiently deployed for management of tree pests.

Overall recovery of dsRNA across both tissue type and exposure interval was 93%. There was a marginal but non-significant decrease in dsRNA presence over time, but we affirmed the persistence of dsRNAs applied as a root soak through 7 days, which is congruent with findings in similar studies. Experiments on *Malus* and *Vitis* species showed that trunk injection of hairpin RNAs lead to reliable recovery at 24 h but recovery diminished quickly through 10 days in leaves distal to the application site; however, when delivered *via* exposed petioles both hairpin and small interfering RNAs accumulated steadily over time (Dalakouras et al., 2018). Hunter et al. (2012) demonstrated recovery of root drench applied dsRNAs in citrus trees through 57 days. Our study terminated after 7 days, but on day 7 dsRNA was still recovered in 83% of stem and 92% of foliar samples having diminished only marginally from 92% and 100% respectively after just 1 day of exposure. Our findings demonstrate the consistent short-term persistence of dsRNAs applied as a root soak in white oak seedlings, though additional studies evaluating extended persistence are needed.

Proteins responsible for processing long dsRNA transcripts into short RNAs (sRNAs) are not unique to plant pests and pathogens. While some insects and fungi possess just two Dicer genes, plants possess a suite of Dicer-like genes (DCLs), each of which are responsible for generating sRNAs vital to cellular processes such as viral defense, chromatin modification, and post-transcriptional gene silencing for regulating physiological pathways (Schauer et al., 2002; Finnegan and Matzke, 2003; Margis et al., 2006). Although plants utilize multiple DCLs, Dicer-like 4 (DCL4), which is generally expressed in the cytoplasm, acts specifically to cleave long dsRNAs into 21-nt siRNAs integral to both cell-cell signaling pathways and antiviral protection (Dunoyer et al., 2005; Pumplin et al., 2016; Kakiyama et al., 2019). Our recovery methods focused solely on intact dsRNA constructs but processing of our dsGFP into siRNA fragments was possible. In *Citrus* sp. siRNAs from processed dsRNAs applied as a root soak persisted within woody plant tissue for at least 57 days and were detected *via* real-time PCR (Hunter et al., 2012; Ghosh et al., 2018). In both *Vitis* and *Malus* spp. Dalakouras et al. (2018) found no siRNA production from hpRNA injected into trunk tissues or absorbed through exposed petioles. Confocal imaging of treated tissues suggested that both trunk-injected and petiole-absorbed hpRNAs remained strictly in xylem tissues, made up of non-protoplasmic vessel elements and tracheids which are believed to be devoid of DCL activity. These discrepancies in siRNA production by woody plants may be due to differences in substrate material (dsRNA vs. hpRNA) and their affinities to degradation by DCL4. Moreover, delivery routes and subsequent RNA dissemination may have impacted which tissues and cell types the treatment RNA interacted with. Processing of our root soak applied dsRNAs into siRNAs could explain some of the diminished recovery of intact treatment material; however future work examining within tissue localization of root soak applied dsRNAs may shed light on the possible processing of these long dsRNAs by plant DCLs.

Our findings provide evidence for rapid dissemination of dsRNAs within woody plant vascular systems. After just 24 h, exogenous dsRNAs applied as a root soak were present in foliar and stem tissue at the seedling apex, where they were detectable for at least 7 days. Previous work in other deciduous trees, both ash (*Fraxinus* spp.) and apple, showed the presence of dsRNA and hpRNA within xylem tissue through confocal imaging (Dalakouras et al., 2018; Pampolini et al., 2020). Both hpRNA and dsRNA are large RNA molecules whose size may initially confine them to the xylem. Phloem is generally believed to be responsible for transporting sRNA molecules *via* bulk flow process from source to sink tissues (Tournier et al., 2006; Melnyk et al., 2011). This rapid dissemination of dsRNAs, likely *via* the plant vascular system, means that shortly after application intact dsRNAs can be found in distal, untreated plant tissues, potentially offering whole plant protection against target pests. However, little work has been done tracing dsRNA molecules in phloem tissues or the subsequent flow of processed dsRNAs as sRNA molecules. Although we focused on evaluating newer, softer tissues, such as foliage and new stems, it is likely that dsRNAs are also present in the older stem and root tissues, at least transiently, as they must pass through the main stem to reach the upper stem and foliage.

Both soil type and environmental conditions may play a role in the degradation of RNAi constructs. Environmental degradation of dsRNAs can occur rapidly, with 90% of dsRNA constructs in soil degraded in as little as 35 h, and 90% degradation of dsRNAs in water in as little as 4 days (Dubelman et al., 2014; Joaquim et al., 2019). However, Fischer et al. (2016) showed that distinct constructs, both hpRNA and dsRNA, degrade at similar rates under identical environmental conditions, suggesting that dissociation is likely independent of sequence, molecular weight, and construct structure. By administering our treatments in these experiments hydroponically, we avoided the complexities of our treatment dsRNAs interacting with a heterogenous soil matrix. Further, the water used for treatment administration was double-distilled and the oak roots were rinsed with dd H_2_O before exposure to dsRNA. Consequently, we were able to evaluate dsRNA uptake, dissemination, persistence, and integration while discounting confounding factors such as soil- or water-associated microbial degradation. Despite these precautions, plant- or surface-associated microbes could potentially have degraded some treatment material before plant uptake. Our experimental seedlings also remained in the dsRNA-water solutions for the duration of the experiment; possible degradation of the naked dsRNAs while in the dsRNA-water solution suggests that uptake was rapid and subsequent dsRNA remained intact within the plant tissue rather than in the water column. While not directly investigated here, understanding the dynamics of dsRNA interactions with soil and soilborne microbes will be integral to deployment of dsRNAs as biopesticides.

### Conclusion and Implications

Our experiment is the first to evaluate the translocation and persistence of exogenously applied dsRNAs delivered *via* hydroponic soaking in white oak and serves to broaden our understanding of dsRNAs in woody plant tissue. Our work demonstrates the presence of dsRNA in young, vulnerable tissues, often the target of defoliation and pathogen attack, suggesting the utility of RNAi technology to provide protection to valuable woody plant tissues. Previous work has evaluated dsRNA distribution and persistence using various delivery techniques (Dalakouras et al., 2018), greater amounts of dsRNA (Hunter et al., 2012), and younger plant material on smaller scales (Pampolini et al., 2020). Our findings prove that dsRNA can be introduced into tree seedlings hydroponically, persist there for at least 7 days, and be recovered and amplified even with only a minute initial dosage. Before RNAi technologies are deployed for tree protection, a deeper understanding of interactions within the woody materials and the environment is needed.

### Future Directions

Our findings provide a deeper understanding of the behavior of dsRNAs when deployed within woody plant tissues and lay the groundwork for future studies investigating RNAi technologies within perennial woody plants on larger scales. Future work investigating the environmental fate of dsRNAs exposed to seedlings in field settings will increase our understanding of the longevity of naked dsRNAs under natural conditions as well as better inform how the soil interface may impact absorption of dsRNAs *via* woody roots. Moreover, only a single dsRNA at a single concentration was evaluated here; therefore, future work investigating multiple dsRNAs of varying length and concentration may better inform how these parameters effect longevity within seeding tissues. Finally, replication with larger plants and higher dosages would be instructive.

## Data Availability Statement

The original contributions presented in the study are included in the article/Supplementary Material, further inquiries can be directed to the corresponding author.

## Author Contributions

ZB conducted the experiments. Both authors conceived the experiments, analyzed and interpreted the results, and prepared and approved the manuscript.

## Conflict of Interest

The authors declare that the research was conducted in the absence of any commercial or financial relationships that could be construed as a potential conflict of interest.

## Publisher’s Note

All claims expressed in this article are solely those of the authors and do not necessarily represent those of their affiliated organizations, or those of the publisher, the editors and the reviewers. Any product that may be evaluated in this article, or claim that may be made by its manufacturer, is not guaranteed or endorsed by the publisher.
