# Supplementary material for: Feasibility of Systemically Applied dsRNAs for Pest-Specific RNAi-Induced Gene Silencing in White Oak
Source: Front Plant Sci. 2022 Mar 16;13:830226. doi: 10.3389/fpls.2022.830226 (PMC8966767; doi:10.3389/fpls.2022.830226)
Supplement: Supplementary file 1 [file Data_Sheet_1.PDF]

Supplementary Material

Supplementary Table S1. dsRNA concentrations and total volume delivered per seedling to attain 16,000 ng treatments.

Replicate	dsRNA	Concentration	Volume Delivered
Replicates 1 & 2	dsGFP	6,558 ng/µL	2.44 µL
Replicates 3 & 4	dsGFP	5,312 ng/µL	3.01 µL

Supplementary Figure S1. Gel of treatment dsGFP (1 µL) used in replicates 1 and 2 (lane 2) and replicates 3 and 4 (lane 3), lane 1 contains 1Kb ladder. Approximate amplicon size listed in base pair (bp).

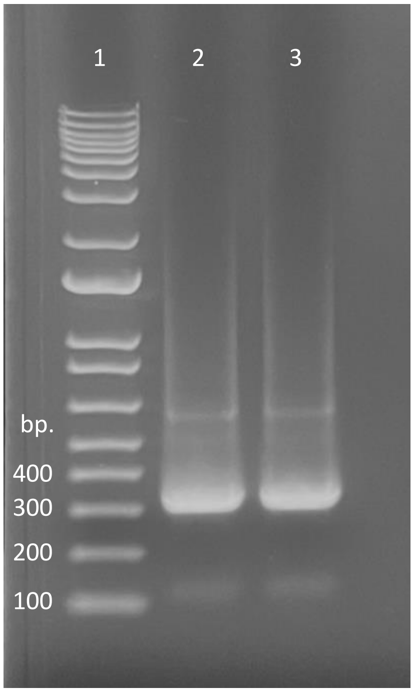

Supplementary Text S1. RNA extraction protocol modified from Chang et al. (1993) and adapted for smaller sample size.

In a 1.5 mL microcentrifuge tube, plant tissue (~200 mg) was combined with 650 µL prewarmed (65 °C) extraction buffer and 12 µL beta-mercaptoethanol, vortexed for 30 s and incubated at 65 °C for 15 min; vortexing for 30 s every 5 min. Next, an equal volume of chloroform/isoamyl alcohol (24:1) was added to the homogenate and vortexed for 30 s three times over 10 min. Following centrifugation at 17200 ×g for 10 min at 4 °C, the supernatant (~400 µL) was transferred to a new 1.5 mL microcentrifuge tube and an equal volume of chloroform/isoamyl alcohol was added. Supernatant and chloroform/isoamyl alcohol were again vortexed for 30 s three times over 10 min, followed by centrifugation at 17200 ×g for 10 min at 4 °C after which the supernatant (~300 µL) was moved to a new microcentrifuge tube. Next a 1/3 volume (~100 µL) of 8M LiCl was added to the mixture, vortexed gently for 5 s, and left to precipitate for 18 h at 4 °C. After precipitation, the mixture was centrifuged at 20800 ×g for 20 min at 4 °C and the supernatant removed and discarded. The visible pellet was resuspended in 500 µL of 50 °C SSTE buffer; resuspension was encouraged with repeated bouts of 30 s vortexing. Once the pellet was completely resuspended, an equal volume of chloroform/isoamyl alcohol was added, and vortexed three times for 30s over 10 min. This mixture was centrifuged at 12000 ×g for 10 min at 4 °C and the supernatant was moved to a final new 1.5 mL microcentrifuge tube; supernatant volume averaged 400 µL, with a maximum of 500 µL. Two volumes of 100% ethanol were added to the supernatant, vortexed for 30 s, and left to precipitate for 2.5 h at –20 °C. After precipitation, the mixture was spun at 20800 ×g for 20 min at 4 °C and the supernatant was discarded. Next, 1 mL of 70% ethanol was added, the mixture was vortexed for 30 s, centrifuged at 20800 ×g for 10 min at 4 °C, after which the supernatant was discarded, and the pellet was allowed to air dry. Once all ethanol was evaporated, the remaining RNA pellet was resuspended in 20 µL of nuclease free water.

Supplementary Table S2. Comparative metrics for logistic regression models for each tissue type where the dependent variable is successful recovery of dsRNA.

	**AIC**		**Efron's Pseudo R^2**	
**Predictor(s)**	**Stem**	**Foliage**	**Stem**	**Foliage**
Null	34.078	18.628	--	--
Time	35.672	18.761	0.0126	0.1123
Height	35.488	14.341	0.0184	0.3781
Root collar diameter (RCD)	35.921	20.110	0.0049	0.0311
RNA yield	35.888	20.328	0.0059	0.0180
Time + Height	37.006	13.674	0.0334	0.5385
Time + Height + RCD	38.970	15.416	0.0345	0.5540
Time + Height + RCD + RNA yield	40.935	16.714	0.0356	0.5962

Supplementary Table S3. Pearson's chi squared test of independence values for logistic regression models predicting the recovery of dsRNA in each oak tissue type.

	Pearson's Chi Squared	
**Predictor**	**Stem**	**Foliage**
Time	$\chi^2_{(1, 47)} = 0.405$, $p = 0.5243$	$\chi^2_{(1, 47)} = 1.87$, $p = 0.1719$
Height	$\chi^2_{(1, 47)} = 0.590$, $p = 0.4426$	$\chi^2_{(1, 47)} = 6.29$, $\mathbf{p = 0.01217}$
RCD	$\chi^2_{(1, 47)} = 0.157$, $p = 0.6921$	$\chi^2_{(1, 47)} = 0.518$, $p = 0.4717$
RNA yield	$\chi^2_{(1, 47)} = 0.190$, $p = 0.6632$	$\chi^2_{(1, 47)} = 0.300$, $p = 0.5840$
